# Arginase Inhibition Reverses Monocrotaline-Induced Pulmonary Hypertension

**DOI:** 10.3390/ijms18081609

**Published:** 2017-07-25

**Authors:** Christian Jung, Katja Grün, Stefan Betge, John Pernow, Malte Kelm, Johanna Muessig, Maryna Masyuk, Friedhelm Kuethe, Bernadin Ndongson-Dongmo, Reinhard Bauer, Alexander Lauten, P. Christian Schulze, Alexander Berndt, Marcus Franz

**Affiliations:** 1 Department of Internal Medicine, Division of Cardiology, Pulmonology and Vascular Medicine, University Hospital Düsseldorf, Heinrich-Heine-University, Düsseldorf 40225, Germany; christian.jung@med.uni-duesseldorf.de (C.J.); malte.kelm@med.uni-duesseldorf.de(M.K.); Johanna.Muessig@med.uni-duesseldorf.de(J.M.); Maryna.Masyuk@med.uni-duesseldorf.de (M.M.); 2 Department of Internal Medicine I, Jena University Hospital, Jena 07747, Germany; Katja.Gruen@med.uni-jena.de (K.G.); Christian.Schulze@med.uni-jena.de (P.C.S.); 3 Department of Angiology, Cardiovascular Center Bad Bevensen, Bad Bevensen 29549, Germany; S.Betge@hgz-bb.de; 4 Department of Medicine, Karolinska Institutet, Karolinska University Hospital, Stockholm 171 76, Sweden; John.Pernow@ki.se; 5 Klinik für Innere Medizin I, Ilm-Kreis-Kliniken Arnstadt, Arnstadt 99310, Germany; Friedhelm.Kuethe@ilm-kreis-kliniken.de; 6 Department of Pharmacology, Institute of Clinical Medicine, University of Oslo, Oslo 0316, Norway; bernadindongs@yahoo.fr; 7 Institute of Molecular Cell Biology, Center for Molecular Biomedicine, Jena University Hospital, Jena 07745, Germany; Reinhard.Bauer@med.uni-jena.de; 8 Department of Cardiology, Charité – Universitätsmedizin Berlin, Berlin 12203, Germany; Alexander.Lauten@charite.de; 9 Institute of Pathology, Jena University Hospital, Jena 07743, Germany; Alexander.Berndt@med.uni-jena.de

**Keywords:** pulmonary hypertension, monocrotaline rat model, arginase, isoforms, inhibition

## Abstract

Pulmonary hypertension (PH) is a heterogeneous disorder associated with a poor prognosis. Thus, the development of novel treatment strategies is of great interest. The enzyme arginase (Arg) is emerging as important player in PH development. The aim of the current study was to determine the expression of ArgI and ArgII as well as the effects of Arg inhibition in a rat model of PH. PH was induced in 35 Sprague–Dawley rats by monocrotaline (MCT, 60 mg/kg as single-dose). There were three experimental groups: sham-treated controls (control group, *n* = 11), MCT-induced PH (MCT group, *n* = 11) and MCT-induced PH treated with the Arg inhibitor Nω-hydroxy-nor-l-arginine (nor-NOHA; MCT/NorNoha group, *n* = 13). ArgI and ArgII expression was determined by immunohistochemistry and Western blot. Right ventricular systolic pressure (RVPsys) was measured and lung tissue remodeling was determined. Induction of PH resulted in an increase in RVPsys (81 ± 16 mmHg) compared to the control group (41 ± 15 mmHg, *p* = 0.002) accompanied by a significant elevation of histological sum-score (8.2 ± 2.4 in the MCT compared to 1.6 ± 1.6 in the control group, *p* < 0.001). Both, ArgI and ArgII were relevantly expressed in lung tissue and there was a significant increase in the MCT compared to the control group (*p* < 0.01). Arg inhibition resulted in a significant reduction of RVPsys to 52 ± 19 mmHg (*p* = 0.006) and histological sum-score to 5.8 ± 1.4 compared to the MCT group (*p* = 0.022). PH leads to increased expression of Arg. Arg inhibition leads to reduction of RVPsys and diminished lung tissue remodeling and therefore represents a potential treatment strategy in PH.

## 1. Introduction

Pulmonary hypertension (PH) is a severe clinical condition associated not only with a reduced quality of life but also with a markedly impaired prognosis. The term PH subsumes a heterogeneous group of disorders resulting in an elevated pulmonary artery pressure (PAP) defined as a mean PAP above 25 mmHg measured by right heart catheterization. The current guidelines describe five clinical groups of PH differing in pathogenesis and especially in treatment options [1,2]. Whereas specific treatment strategies exist for pulmonary arterial hypertension (PAH) and chronic thromboembolic PH (CTEPH) by using prostanoids, endothelin receptor antagonists, phosphodiesterase inhibitors or a soluble guanylate cyclase agonist (only approved treatment option for group 4 of PH = CTEPH), therapy is limited to the treatment of the underlying disease and symptom control for the remaining groups, especially PH due to left heart disease and PH due to pulmonary disease/hypoxia, [3]. In either case, until now there are no curative treatment strategies to stop or even reverse the process of pulmonary vascular remodeling which, together with vasoconstriction and thrombosis, are the main pathophysiological building blocks of PH irrespective of the aetiology [4].

The main function of the enzyme arginase (Arg) is the conversion of l-arginine to l-ornithine and urea [5,6]. As a result of this, Arg competes with nitric oxide (NO) synthase for their common substrate l-arginine. By this mechanism, Arg plays an important role in the regulation of endothelial function and vessel wall remodeling both in systemic and pulmonary hypertension. Thus, the enzyme is of great interest as a potential therapeutic target to causally influence the development of PH [7]. Arg exists in the two distinct isoforms Arg I and Arg II, which show species-specific differences both in tissue distribution and cellular/subcellular localization. Both isoforms are induced by pathologic stimuli such as hypoxia [6] and mediate several processes resulting in an impaired vascular cell function and detrimental vessel wall remodeling, which, in the case of PH, leads to an elevated PAP [7,8,9,10,11]. The induction and perpetuation of vessel wall remodeling by Arg is not limited to its reciprocal regulation of NO formation but is also due to its capability to enhance proliferation of vascular smooth muscle cells and endothelial cells [10,12,13,14]. Moreover, Arg stimulates synthesis and extracellular deposition of collagen [15]. Taken together, the main effects of Arg I and Arg II are the mediation of proliferative, fibrotic and inflammatory reactions within the complex process of vascular remodeling [7]. On the other hand it is important to point out that the up-regulation of Arg is not restricted to cardiovascular disorders but is also present in physiological processes like wound healing or as a constituent of the urea cycle [16,17].

Previous studies have indicated a role of Arg I and Arg II in PH. Arg inhibition reduced inflammation, airway remodeling and right ventricular hypertrophy in a guinea pig model of chronic obstructive pulmonary disease [18]. Arg expression is increased and Arg inhibition prevents PAH development in a rat model of PH induced by intermittent hypoxia suggesting a crucial pathological role of the enzyme [19]. Human pulmonary artery smooth muscle cells could be evidenced to be an important cell phenotype within the process of hypoxia-induced PH since Arg inhibition significantly attenuated proliferative capacity in these cells [20]. Arg inhibition could also prevent bleomycin-induced PH, vascular remodeling and collagen deposition in neonatal rats [21]. Another interesting study in a rat model of monocrotaline-induced PH could show that deactivation of the sympathetic nervous system by cervical ganglion block resulted in attenuation of the progression of PH via the NO and Arg pathways [22]. In a very recent small human study in patients with PAH it could be demonstrated that there is an increased breakdown of arginine by Arg affecting NO synthesis compared to healthy controls. These findings highlight the role of Arg in PH development [23]. Further data on PH as a consequence of hemolysis due to the increased release of Arg 1 from damaged erythrocytes speak well for a functional role of the enzyme in PH pathogeneses. This could be shown in patients suffering from sickle cell disease or thalassemia as well. [24,25] Since two distinct Arg isoforms exist, the question arises which isoform is of predominant functional relevance in PH development. From recent studies the following is known: Both, Arg I and Arg II are up-regulated in mouse models of hypoxia induced PH [26]. In the human system, Arg II but not Arg I was detected in pulmonary arterial endothelial cells [27]. Most studies reporting on the crucial role of Arg in PH development in animal models, identified Arg II as the most relevant isoform [10,12,13,28,29,30]. In this context the phenomenon of species specificity of Arg isoform expression must be taken into account [7]. Since most studies have been performed in rodent animal models, there is a limited transferability of the results into the human system.

There is growing evidence that pharmacological Arg inhibition [31] is a promising novel tool to therapeutically intervene in disorders associated with PH development. A well established and widely accepted animal model of PH is the rat model of monocrotaline-induced PH [32]. The majority of preclinical studies on treatment of group 1 PH (PAH) have been performed using this model. Despite several limitations of the model, there is no better alternative until now qualifying it as state of the art in preclinical testing of novel agents for specific PH treatment [33]. Aims of the study: Our current study was aimed to differentially investigate for the first time the structural changes in lung tissue remodeling in the classical model of monocrotaline induced PH in relation to Arg isoform expression and differential tissue distribution as well as to elucidate the effectiveness of non-selective Arg inhibition as novel treatment approach in PH.

## 2. Results

### 2.1. Clinical and Haemodynamic Characterization of the Different Experimental Groups

**Figure 1:**
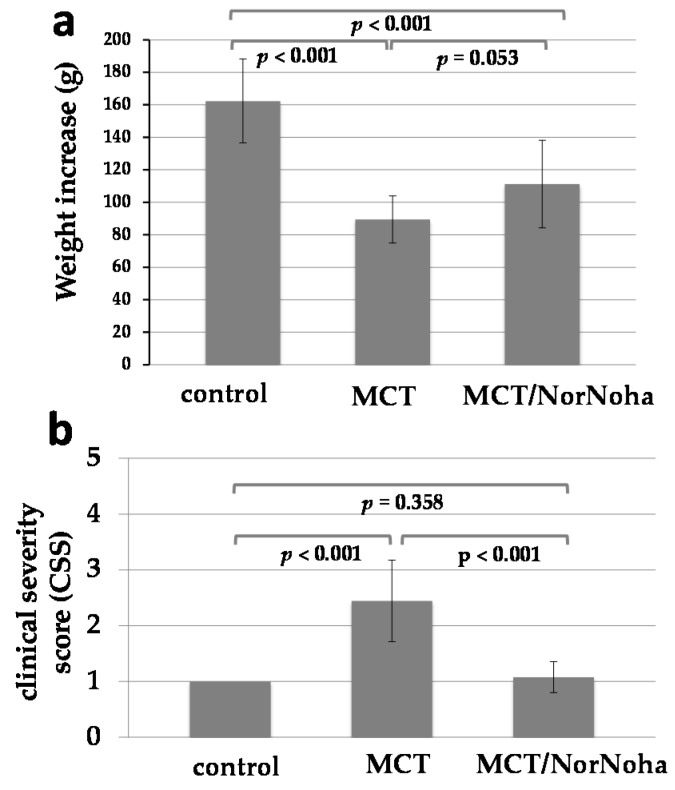
Graphical presentation of body weight increase (**a**) and clinical severity score (**b**) in the three experimental groups: control = sham-treated animals (*n* = 11); MCT = monocrotaline induced PH (*n* = 11); MCT/nor-NOHA = monocrotaline induced PH treated with nor-NOHA (*n* = 13). Data are presented as means and standard deviation (SD).

Two of the 35 rats included in this study died throughout the experiments for unknown reasons. These rats belonged to the MCT group. All the other 33 rats showed a continuous increase in body weight but the level was different between the groups: control group (*n* = 11, mean weight increase 162 ± 26 g) > MCT/nor-NOHA group (*n* = 13, mean weight increase 111 ± 27 g) > MCT group (*n* = 9, mean weight increase 89 ± 14 g). The comparison of the different groups including *p*-values is given in Figure 1a. Clinical state of health of the animals was frequently assessed as described above (clinical severity score). As depicted in Figure 1b, MCT treated rats had a reduced clinical state of health compared to the control group, nor-NOHA treatment was associated with both improved weight increase and higher clinical severity scores compared to the MCT group (Figure 1a,b).

Differential protein expression analysis of Arg I and Arg II in rat lung tissue of MCT induced pulmonary hypertension.

### 2.2. Immunohistochemistry

Arg I and Arg II expression could be detected not only in diseased but also in healthy lung tissue. As already known from a detailed analysis of Arg I und Arg II tissue expression in healthy rat organs performed by Choi and co-workers in 2012 [34], both enzymes show a moderate expression in alveolar macrophages. Arg I is additionally expressed in the epithelium of the bronchioles and the alveoli at a moderate level. Arg II shows a weak expression in the epithelium of the bronchioles and a minimal expression in the alveoli. These expression patterns could be, in general, confirmed also in the healthy rat lung tissue obtained from our animal model. Moreover, especially for Arg II, we could find a weak positivity of endothelial cells in peribronchial arteries, which has not been described before.

**Figure 2:**
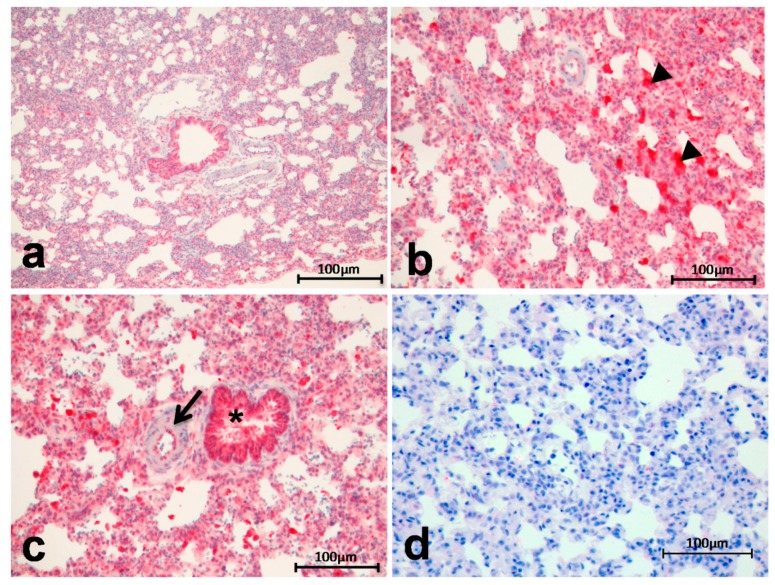
Arg I showed an increased expression and tissue deposition in induced pulmonary hypertension (IPH) rats (**b**–**c**) compared to the controls (**a**) enhanced staining positivity in the lung parenchyma and in alveolar macrophages ((**b**), arrowheads = alveolar macrophages) and mild to moderate positivity in endothelial cells of peribronchial arteries ((**c**), arrow); staining intensity of the epithelium of the bronchioles is more or less equal to that occurring in normal tissue ((**c**), *); negative control for Arg I staining (**d**).

Arg I showed an increased expression and tissue deposition in diseased lung tissue of IPH rats (MCT group) compared to healthy organs (control group) (Figure 2). There is an enhanced staining positivity in the lung parenchyma and, especially, in the alveolar macrophages (Figure 2b, arrowheads = alveolar macrophages). Moreover, endothelial cells of peribronchial arteries show a mild to moderate positivity (Figure 2c, arrow) compared to healthy lung tissue (Figure 2a). The staining intensity of the epithelium of the bronchioles is more or less equal to that occurring in normal tissue (Figure 2c, *). Figure 2d shows the negative control for Arg I staining (Figure 2d).

**Figure 3:**
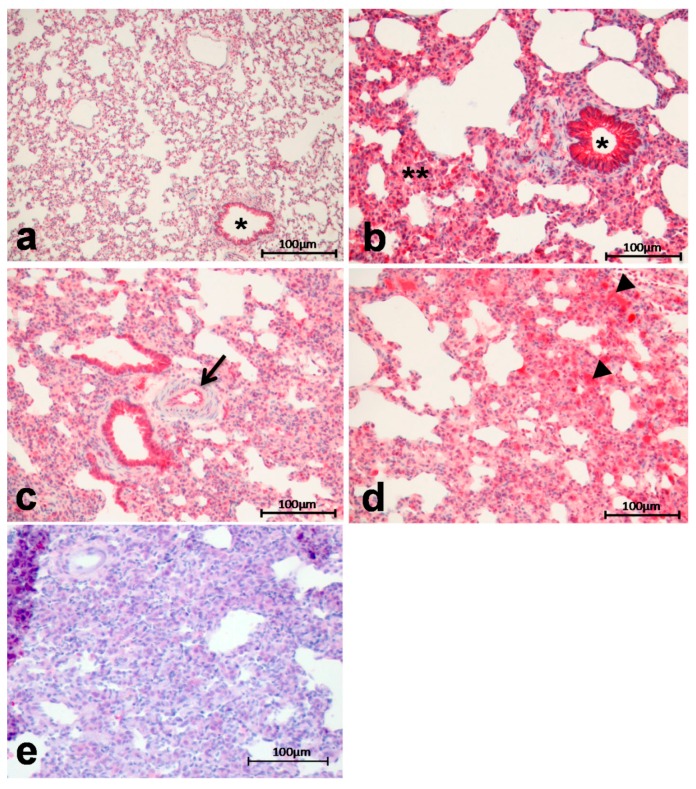
Arg II exhibited a distinct increase in expression and tissue deposition in IPH rats (**b**–**d**) compared to controls ((**a**), * = bronchiolus) in particular in the lung parenchyma (**) and the epithelium of the bronchioles (*) (**b**), the endothelium of peribronchial arteries ((**c**), arrow) and in alveolar macrophages ((**d**), arrowheads = alveolar macrophages); negative control (**e**).

Arg II exhibited a distinct increase in expression and tissue deposition in diseased lung tissue from IPH rats (MCT group) compared to healthy organs (control group) (Figure 3), in particular in the lung parenchyma and the epithelium of the bronchioles (Figure 3b), the endothelium of peribronchial arteries (Figure 3c) and in alveolar macrophages (Figure 3d). Figure 3e shows the negative control for Arg I staining (Figure 3e).

### 2.3. Western Blot Analysis

To confirm the expression of Arg protein including a semi-quantitative analysis, we performed Western blot analysis of Arg I and Arg II.

**Figure 4:**
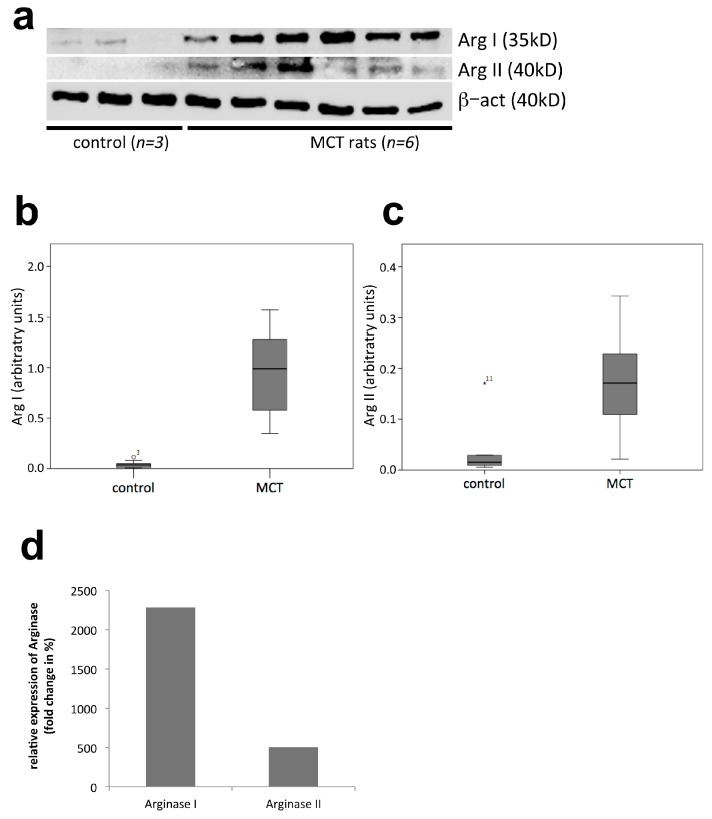
Western Blot and semi-quantitative analysis of Arginase I and II expression in PH rats (MCT group, *p* = 8) compared to controls (*p* = 9): (**a**) representative Western Blot experiment showing increased expression of arginase I and arginase II in MCT treated compared to sham-treated control rats (β-actin served as housekeeping protein); (**b**–**d**) software-based densitometric semi-quantitative analysis of arginase I and II expression normalized to β-actin. Data are presented as means and SD.

A representative Western blot membrane presented in Figure 4a shows increased expression of Arg I and, to a lesser extent, Arg II in MCT-treated compared to sham-treated control rats.

Semi-quantitative analysis revealed a significantly increased expression in the MCT compared to the control group both for Arg I (22.7 fold; *p* = 0.001; Figure 4b,d) and for Arg II (5.1-fold; *p* = 0.004; Figure 4c,d).

Treatment effects of the Arg inhibitor nor-NOHA in MCT induced pulmonary hypertension.

### 2.4. Haemodynamics

**Figure 5:**
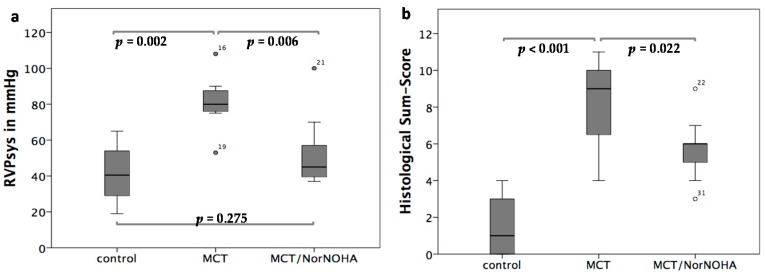
(**a**) Comparison of haemodynamic treatment effects of the Arginase inhibitor nor-NOHA: significant increase of RVPsys values in the MCT compared to the control group (*p* = 0.002), decreased RVPsys values in the MCT/nor-NOHA compared to the MCT group (*p* = 0.006) and non-significantly elevated RVPsys values between the MCT/NorNoha and the sham-treated control group (*p* = 0.275). Data are presented as means and SD; (**b**) comparison of treatment effects of the Arginase inhibitor nor-NOHA with respect to lung tissue remodelling: the sham-treated control group showed healthy lung tissue, the MCT group exhibited distinct signs of lung damage (*p* < 0.001 compared to the control group), which were diminished in the MCT/nor-NOHA group (*p* = 0.022 compared to the MCT group). Data are presented as means and SD.

When comparing the RVPsys values in the three different experimental groups, a significant increase in RVPsys in the MCT (RVPsys = 81.14 ± 16.65 mmHg; heart rate = 325 ± 22 bpm) compared to the control (RVPsys = 41.2 ± 15.86 mmHg; heart rate = 308 ± 29 bpm) group could be demonstrated (*p* = 0.002). In the MCT/nor-NOHA (RVPsys = 52.09 ± 19.10 mmHg; heart rate = 310 ± 14 bpm) group, RVPsys were significantly decreased compared to the MCT group (*p* = 0.006) and did not differ from that of the sham-treated control group (*p* = 0.275) (Figure 5a). There were no significant differences with respect to the heart rate of the animals in the three experimental groups (*p* = 0.123).

### 2.5. Histological Evaluation of Lung Tissue Remodeling Using a Sum-Score System

The sham-treated control group showed healthy lung tissue (sum-score 1.59 ± 1.56), the MCT group exhibited distinct signs of lung damage (sum-score 8.17 ± 2.42; *p* < 0.001 compared to the control group), which were diminished in the MCT/nor-NOHA group (sum-score 5.77 ± 1.42; *p* = 0.022 compared to the MCT group) as shown in Figure 5b.

## 3. Discussion

The current study was focused on the investigation of the differential protein expression of the Arg isoforms I and II in the rat model of MCT induced PH and to evaluate the effect of the Arg inhibitor nor-NOHA. The animal model was chosen because it is, besides models of hypoxia induced PH, a well-established model, which has been used for a long time to investigate PH both, in terms of mechanistic studies as well as preclinical testing of novel treatment strategies. As for any other PH animal model available, it has several limitations and some concerns should be kept in mind when interpreting the results obtained with this model [32,33]. Against the background of the fact that most studies investigating the role of Arg in PH development as well as effects of enzyme inhibition were performed using models of hypoxia induced PH [19,20,28,35], it is of certain scientific interest to elucidate these processes in the MCT model as performed in the current study. Protein expression and tissue distribution analysis clearly showed an increased expression of both, Arg I and Arg II in induced PH compared to controls. Both enzymes revealed typical and distinguishable spatial associations to several lung tissue components or certain cell types. The obtained results mainly go in line with the findings of Choi and co-workers who described tissue expression of Arg I and Arg II in healthy rat organs in a sophisticated way in Sprague–Dawley rats, the same strain as used in this study for the MCT model [34]. On the quantitative level, the current study could show that there is a relevant increase in protein expression of both, Arg I and Arg II. While for Arg II, a 5.1-fold increase was observable, Arg I revealed a 22.7-fold increase. The finding is novel and its discussion against the background of the available literature is challenging due to most in vivo studies in animal models, predominantly models of hypoxia induced PH, focusing on Arg II, while not comparatively looking for Arg I or even differentiating between the isoforms [12,18,19,20,21,22,29,36]. This might be for the following reasons: first, Arg isoform expression depends on both, the species used for an animal model as well as the pathologic stimulus leading to Arg up-regulation (reviewed in [7]). Second, in human PAH, Arg II seems to be of predominant significance compared to Arg I at least when referring to the available studies, which are hitherto rare [26,27]. For those reasons, especially species specificity of Arg isoform expression might explain our current findings in the rat model. For a variety of cell types, in particular smooth muscle cells and endothelial cells, it could be evidenced that Arg I is the predominantly expressed isoform in rats [8,9,37,38] whereas it is Arg II in humans [12,14,36,39,40,41,42].

In correspondence with our finding concerning a certain role of Arg I in induced PH, Cowburn and co-workers observed a HIF2α enhancement triggered by chronic hypoxia in a mouse model resulting in an up-regulation of its downstream target Arg I with the consequence of a dysregulated NO homeostasis [28]. There are only few in vivo studies dealing with the impact of Arg in rat models of PH [19,21,22,35]. From these, only one study used the MCT model [22], two studies used hypoxia as pathologic stimulus [19,35] and one study bleomycin [21]. Neither of these studies discriminated between Arg isoforms. Taken together the findings of the first part of our study it has to be noted that in the MCT rat model of PH Arg I is the predominantly expressed isoform. This is likely at least in part due to species-specific aspects. The question if there is a specific functional reason for Arg I instead of Arg II must be the objective of further studies.

By treatment with the non-selective Arg inhibitor nor-NOHA, we could show two main effects: first, a significant reduction of RVPsys to levels of untreated controls and second, a reverse lung tissue remodelling. Against the background of Arg isoform expression analysis we hypothesize that these effects are mediated by Arg I inhibition. But, since also Arg II was mildly upregulated in our model, only a selective Arg inhibitor would definitely answer the question, which isoform truly mediates therapy response. Unfortunately, such an inhibitor is not available by now.

The data obtained in this study clearly demonstrate a reduction of RVPsys measured invasively by Arg inhibition. This finding goes in line with the observations of Nara and co-workers showing a reduction of hypoxia induced PH in response to Arg inhibition using nor-NOHA [19]. Similar results could be obtained by Jiang and co-workers [35]. By applying the Arg inhibitor amino-2-borono-6-hexanoic acid, bleomycin induced PH could be prevented in a further study in neonatal rats [21]. A very interesting recent study focused on the effects of Arg inhibition in a mouse model of sickle cell disease and could clearly show a reversal of endothelial dysfunction, vascular stiffness and PH by improving NO bioavailability [43].

In summary of our results and the limited number of studies available from the literature, Arg inhibition seems to be a promising novel strategy in the prevention or treatment not only of group 1 PH (PAH) but likely also in other groups, in particular group 3 (lung disease or hypoxia associated PH).

To our very best knowledge, this is the first study that investigated the effects of Arg inhibition in lung tissue remodelling in PH. We used a sum-score system comprising all relevant features of PH associated lung alterations on the histological level including pulmonary vascular remodelling. This scoring system has been recently developed by our group [44]. As described above, nor-NOHA treatment (MCT/nor-NOHA group) resulted in a significant reduction of the sum-score levels compared to the MCT group. Although not applying a detailed lung tissue remodelling score, there are some recent studies demonstrating positive effects of Arg inhibition on pulmonary artery remodelling and smooth muscle cell proliferation, which goes in line with our current findings. Thus, we hypothesize that, besides haemodynamic effects, also a reverse lung tissue and pulmonary vascular remodelling is initiated by Arg inhibition possibly leading to disease reversal also on the causative level. This would certainly be of great clinical impact and should therefore necessarily be the subject of further functional studies.

## 4. Material and Methods

### 4.1. Animal Model of Induced Pulmonary Hypertension and Treatment Protocol

Sprague–Dawley rats (200–250 g) were obtained from Charles River (Sulzfeld, Germany) and allowed to acclimatize for 7 days with ad libitum access to food and water and exposure to controlled light/dark cycles before starting experiments. All experiments were carried out in accordance to the National Institute of Health Guidelines for the Care and Use of Laboratory Animals (8th edition; available online: https://www.ncbi.nlm.nih.gov/books/NBK54050/) and to the European Community Council Directive for the Care and Use of Laboratory Animals of 22 September 2010 (2010/63/EU; available online: http://ec.europa.eu/environment/chemicals/lab_animals/legislation_en.htm). The study protocol was approved by the appropriate State Office of Food Safety and Consumer Protection (TLLV, Bad Langensalza, Germany; local registration number (identification code): 02-004/14, approval date: 14 March 2014). 

The 35 rats were divided into three experimental groups: 11 rats were injected with NaCl (300 µL) subcutaneously and were used as controls (control group), 11 rats were treated with monocrotalin (MCT, Carl Roth, Karlsruhe, Germany) alone to induce PH (MCT group) and 13 rats were treated with MCT followed by a treatment with the non-selective arginase inhibitor Nω-hydroxy-nor-l-arginine (nor-NOHA; Bachem, Bubendorf, Switzerland) (MCT/nor-NOHA group). MCT was administered by subcutaneous injection of a single dose of 60 mg/kg body weight in a volume of 300 µL. Nor-NOHA was administered by intra-peritoneal injection in a dose of 100 mg/kg body weight once daily from day 14 to day 28 post injectionem (p.i.) For the prevention of secondary infections or inflammatory lung-alteration, rats received Enrofloxacin (Baytril, WDT, Garbsen, Germany) 2.5% ad water from day 2 to 15. On day 28 p.i., rats were anaesthetized intraperitoneally and haemodynamic measurements were performed as described below directly before rats were sacrificed in deep anaesthesia and analgesia. Organs were excised and immediately shock frozen in liquid nitrogen and stored at −80 °C or formalin-fixed and paraffin-embedded until further analysis. To carefully monitor clinical well-being of the animals, rats were weighed and examined daily and the state of health was evaluated by applying a clinical severity score (CSS). This score estimates the following five parameters and ranges from 1 to 5 for each of them: weight development; spontaneous activity; reaction to exogenous stimuli, posture / social behavior and breathing (1 = no signs of illness; 2 = low-grade impairment; 3 = mild-grade impairment; 4 = high grade impairment; 5 = dead).

### 4.2. Haemodynamic Measurements

Right heart catheterization (RHC) was performed using a 1.4F micro conductance pressure-volume catheter (model SPR-839; Millar Instruments Inc, Houston, TX, USA) inserted via the right jugular vein. Right ventricular blood pressure and, if technically feasible, also pulmonary arterial blood pressure traces were continuously registered in closed chest animals. Analysis of pressure curves was performed using a PowerLab system (ADInstruments Ltd., Sydney, Australia) connected to the catheter.

### 4.3. Histological Evaluation

A total of 4 µm sections of the formalin-fixed and paraffin-embedded lung tissue were subjected to H&E-staining (HE). Analysis of the extent of lung tissue damage was performed using a sum-score system (ranging from 0 to 12 points) including the most relevant histopathological features occurring in PH, namely atelectasis area, emphysema area, media hypertrophy of peribronchial arteries, perivascular cellular edema of peribronchial arteries and media hypertrophy of small arteries as described by our group recently. In detail, the semi-quantitative score was assessed as follows: parameter 1 = atelectasis area (area in % of atelectasis related to total area of tissue section): not detectable = 0 points, <30% = 1 point, ≥30% = 2 points; parameter 2 = emphysema area (area in % of emphysema related to total area of tissue section): not detectable = 0 points, <30% = 1 point, ≥30% = 2 points; parameter 3 = media hypertrophy of peribronchial arteries (cellular hypertrophy of the tunica media of arteries spatially associated to bronchial structures): not detectable = 0 points, weak = 1 point, moderate = 2 points, severe = 3 points; parameter 4 = perivascular cellular edema of peribronchial arteries (cellular edema locates in the perivascular region around peribronchial arteries): not detectable = 0 points, detectable = 2 points; parameter = media hypertrophy of small arteries (cellular hypertrophy of the tunica media of small arteries showing no spatial association to bronchial structures): not detectable = 0 points, weak = 1 point, moderate = 2 points, severe = 3 points. The maximum sum-score value is 12 points [44].

### 4.4. Immunohistochemistry

For immunohistochemical detection of Arg I and Arg II, 4 µm sections of formalin-fixed/paraffin-embedded lung tissue were deparaffinized and subjected to antigen-retrieval with citrate-buffer (pH 9.0) for 30 min heated to 95 °C. Non-specific staining due to endogenous biotin was inhibited by applying the Dako Biotin Blocking System (Dako Deutschland GmbH, Hamburg, Germany). Arg I antibody (dilution 1:100; clone H-52, rabbit polyclonal antibody, Santa Cruz Biotechnology, Dallas, TX, USA) and Arg II antibody (dilution 1:125; clone H-64, rabbit polyclonal antibody, Santa Cruz Biotechnology, Dallas, TX, USA) were applied over night at 4 °C. In the next step, sections were incubated with a biotin-conjugated donkey anti- rabbit antibody (1:200, Jackson ImmunoResarch, West Grove, Chester County, PA, USA) and furthermore with an AP-conjugated streptavidin-biotin-antibody(1:75, SouthernBiotech, Birmingham, AL, USA). Detection of bound antibodies was then performed using the Chromogens of the DAKO-Real Detection System (AP/Red; Dako Deutschland GmbH, Hamburg, Germany). Immunostained sections were counterstained with haematoxylin. As negative control, the antibodies were replaced by nonimmune serum.

### 4.5. Protein Isolation and Western Blotting

Proteins were isolated from shock frozen lung tissue. After tissue maceration in liquid nitrogen, triton lysis buffer (50 mM 4-(2-hydroxyethyl)-1-piperazineethanesulfonic acid (HEPES), pH 7.5, 150 mM NaCl, 1.5 mM MgCl_2_, 1 mM Ethylenediaminetetraacetic acid (EDTA), 1% Tritonx 100, 1% Sodiumdeoxychoacid, 0.1% Sodium dodecyl sulfate (SDS), 10% glycin, 10 mM Sodiumpyrophosphate, 1 mM 1,4-Dithiothreitol (DTT) containing a protease inhibitor cocktail (complete ULTRA Tablets; Roche Diagnostics GmbH, Mannheim, Germany) was added (100 mg tissue/1 mL lysis buffer) and allowed to incubate for 30 min on ice. In a next step, lysates were centrifuged at 14,000 rpm for 20 min at 4 °C. The supernatant was transferred to new tubes and protein concentration was determined by the Pierce BCA Protein-Assay Kit (Thermo Fisher Scientific, Waltham, MA, USA). Samples of the supernatants (controls and monocrotalin-treated rats) containing 20 µg protein were diluted 1:2 in loading buffer and used for SDS-PAGE. After electrophoresis, proteins were transferred to a polyvinylidene difluoride membrane and blocked for 1 h in TBS/0.1% Tween (TBS-T) containing 5% nonfat dried milk.

Membranes were then assayed for β-actin (dilution 1:1000, clone C4, mouse monoclonal antibody, Merck Millipore, Billerica, MA, USA) as housekeeping protein and Arg I (dilution: 1:100, clone 19/Arginase I, mouse monoclonal antibody, BD Transduction Laboratories, Franklin Lakes, NJ, USA) and Arg II (dilution 1:100, clone C-3, mouse monoclonal antibody, Santa Cruz Biotechnology, Dallas, TX, USA). The membranes were incubated with the primary antibodies overnight at 4 °C, washed three times with TBS-T, and subsequently treated with a peroxidase conjugated donkey-anti-mouse IgG secondary-antibody (Jackson Immuno Research Lab., West Grove, PA, USA) for 1 h at room temperature. After washing with TBS-T, the protein bands were visualized by chemiluminescence (BM Chemiluminescence Western Blotting Kit (mouse/rabbit), Roche Diagnostics GmbH, Mannheim, Germany) and quantified by densitometry analysis using the ImageJ software, (National Institutes for health (NIH), available online: http://rsb.info.nih.gov/ij/download.html).

### 4.6. Statistics

Statistical analyses were performed with IBM SPSS statistics, version 22.0 (IBM Inc., North Castle, NY, USA). Data are expressed as mean ± standard deviation (SD). Kruskal-Wallis Test was used to test for significant differences between different experimental groups. A *p*-value < 0.05 defined statistical significance.

## 5. Conclusions

We could provide novel data concerning the role of Arg isoforms and Arg inhibition in an appropriate animal model of PH. Besides a striking reduction of RVPsys, we could show for the first time that Arg inhibition partially reverses lung tissue remodeling. Integration of our findings and further recent studies leads to the suggestion that this novel treatment strategy might not only be promising for group 1 but also for other groups of PH, for which there are no specific therapy options available until now.

## Abbreviations

Arg	Arginase
CTEPH	Chronic Thromboembolic Pulmonary Hypertension
MACI	Macitentan
MCT	Monocrotaline
Nor-NOHA	Nω-hydroxy-nor-l-arginine
p.i.	post injectionem
PAH	Pulmonary Arterial Hypertension
PAP	Pulmonary Artery Pressure
PH	Pulmonary Hypertension
RVPsys	Systolic Right Ventricular Pressure
